# The REPLUMLESS Transcription Factor Controls the Expression of the *RECEPTOR-LIKE CYTOPLASMIC KINASE VI_A2* Gene Involved in Shoot and Fruit Patterning of *Arabidopsis thaliana*

**DOI:** 10.3390/ijms25148001

**Published:** 2024-07-22

**Authors:** Erzsébet Kenesi, Orsolya Beöthy-Fehér, Réka Szőllősi, Ildikó Domonkos, Ildikó Valkai, Attila Fehér

**Affiliations:** 1 Institute of Plant Biology, HUN-REN Biological Research Centre, H-6726 Szeged, Hungary; kenesi.erzsebet@brc.hu (E.K.); beothy-feher.orsolya@brc.hu (O.B.-F.); domonkos.ildiko@brc.hu (I.D.); valkai.ildiko@brc.hu (I.V.); 2 Doctoral School in Biology, Faculty of Science and Informatics, University of Szeged, H-6726 Szeged, Hungary; 3 Department of Plant Biology, Faculty of Science and Informatics, University of Szeged, H-6726 Szeged, Hungary; szoszo@bio.u-szeged.hu

**Keywords:** phyllotaxis, replum, stem thickness, BELLRINGER transcription factor, vascular bundles, ROP GTPase-activated kinase

## Abstract

The promoter of the *RECEPTOR-LIKE CYTOPLASMIC KINASE VI_A2* (*RLCK VI_A2*) gene contains nine binding sites for the REPLUMLESS (RPL) transcription factor. In agreement, the expression of the kinase gene was strongly downregulated in the *rpl-4* mutant. Comparing phenotypes of loss-of-function mutants, it was revealed that both genes are involved in stem growth, phyllotaxis, organization of the vascular tissues, and the replum, highlighting potential functional interactions. The expression of the *RLCKVI_A2* gene from the constitutive 35S promoter could not complement the *rpl-4* phenotypes but exhibited a dominant positive effect on stem growth and affected vascular differentiation and organization. The results also indicated that the number of vascular bundles is regulated independently from stem thickness. Although our study cannot demonstrate a direct link between the RPL and *RLVKVI_A2* genes, it highlights the significance of the proper developmental regulation of the *RLCKVI_A2* promoter for balanced stem development.

## 1. Introduction

In plants, Rho-of-plant (ROP) GTPases play a central role in morphogenesis by regulating cell shape, polarity, hormonal responses, and defense mechanisms [1]. The signaling activity of Rho-type GTPases depends on their GTP-bound conformation that facilitates their binding to downstream effector proteins. Recent studies have demonstrated the link of ROPs via their positive regulator guanine nucleotide exchange factors to receptor kinase signaling, but our knowledge of the downstream effectors in these signaling pathways is still limited [2,3]. As ROP effectors, a specific class of receptor-like cytoplasmic kinases (RLCKs) has been identified in several plant species [2,4,5,6,7,8]. In *Arabidopsis thalina*, these kinases belong to group A of the RLCK VI class (RLCKVI_A1-7) [9] and have been implicated in the control of plant growth, morphogenesis, and defense [2,5,6,7,10].

Intriguingly, the *Arabidopsis RLCKVI_A2* gene contains multiple REPLUMLESS (RPL) homeobox transcription factor (TF) binding sites in its promoter. The RPL TF, also known as VAAMANA, PENNYWISE, LARSON, or BELLRINGER, has diverse roles in plant development, including the control of shoot meristem structure and integrity [11,12,13,14]; the spatial arrangement of lateral organs (phyllotaxis of leaves, flowers, and siliques) [15,16,17]; vascular differentiation [13]; and flower [18,19,20,21] and fruit [22,23] development.

Among the RPL-regulated processes, ROP signaling has been associated with those that depend on cell wall composition, namely the secondary cell wall development during vascular differentiation [13,24] and the pectin methylesterase activity-dependent phyllotactic arrangement of lateral organs developing from the shoot meristem [15,25]. These observations prompted us to investigate the possible role of the ROP-activated RLCKVI_A2 kinase in these processes downstream of the RPL TF. It was first verified that the RPL TF is required for *RLCKVI_A2* expression. Next, the *RLCKVI_A2* cDNA was expressed under the control of the 35S promoter in wild-type, *rpl4*, and *rlckvi_a2* plants. Morphological and histological comparisons revealed that the RPL TF and the RLCKVI_A2 kinase are involved in similar processes. Interestingly, the *rpl4* and *rlckvi_a2* mutants often exhibited contrasting phenotypes. Furthermore, the strong ectopic expression of the kinase gene had the most profound impact on the investigated processes independent of the genetic background, suggesting that the tight control of *RLCKVI_A2* expression by its own promoter is required for the correct organization/development of the investigated tissues/organs.

## 2. Results

### 2.1. The RPL Homeobox TF Controls RLCKVI_A2 Gene Expression

The *RLCKVI_A2* gene has a diversity of TF binding sites in its promoter. The gene is likely under the control of a range of transcription factors belonging to the bZIP, GATA, MYB or MYB-related, LFY, ABI3, WRKY, E2F/DP, and homeobox families (Table S1). The homeobox family is represented by nine binding motifs for the RPL TF.

**Figure 1:**
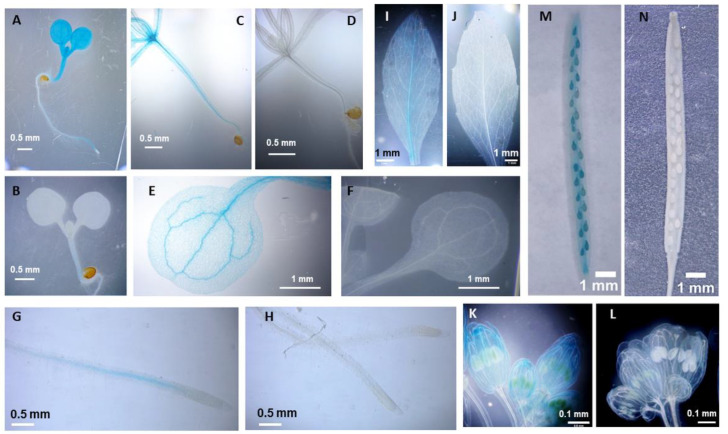
Comparison of the expression of the *At2G18890* (RLCKVI_A2) promoter driving the *GUS* marker gene in wild-type (**A**,**C**,**E**,**G**,**I**,**K**,**M**) and *rpl-4* mutant (**B**,**D**,**F**,**H**,**J**,**L**,**N**) genetic backgrounds.

To verify the significance of RPL in the regulation of *RLCKVI_A2* expression, a fragment of the gene containing 928 bp upstream and 512 bp downstream regions from its start codon was fused to the β-glucuronidase (GUS) reporter gene. In this way, the chimeric gene contained the first exon, the first intron, and a short fragment of the second exon of the *RLCKVI_A2* gene in addition to the promoter. The predicted translation product thus has the first 147 amino acids of the kinase in fusion with the GUS enzyme (Figure S1A). This chimeric gene was introduced into wild-type and *rpl-4* mutant *Arabidopsis* plants. The *rpl-4* mutant has been previously shown to lack *RPL* transcripts [26]. In young wild-type seedlings, the promoter expressed the fusion protein in all organs with strong signals in the shoot, especially in its meristematic region and the vasculature, but no signal in the root meristem (Figure 1A–H). In eight-week-old flowering plants, the expression was strongest in the veins of leaves, the petals and carpels of flowers, and the siliques (Figure 1I–L). In the *rpl-4* genetic background, the expression of the construct was barely detectable, indicating that RPL is required for proper *RLCKVI_A2* expression in all organs from the seedling stage to maturity.

### 2.2. The RLCKVI_A2 and the RPL Genes Are Involved in Similar Processes

**Figure 2:**
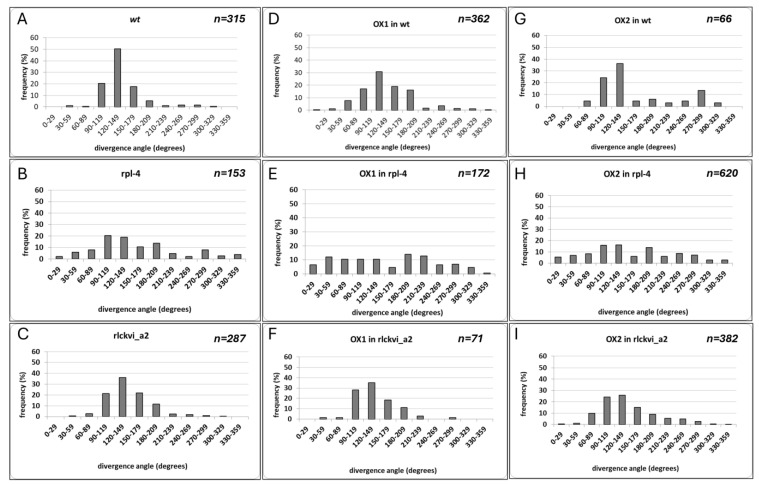
The phyllotactic pattern of siliques of wild-type (wt) (**A**,**D**,**G**), *rpl-4* (**B**,**E**,**H**), and *rlckvi_a2* (**C**,**F**,**I**) mutants without (**A**–**C**) and with (**D**–**I**) ectopic expression (OX1 and OX2) of the *RLCKVI_A2* gene under the control of the 35S promoter, respectively, is shown. The divergence angles of two successive siliques on the stem were determined for a minimum of ten T4 generation plants at the developmental stage 8 [28]. The distribution of measured angles falling into the indicated angle size categories is shown according to [29].

The T-DNA insertion mutant *rlckvi_a2* has previously been reported to control cell and hypocotyl elongation, as well as overall plant growth [10]. Here, we report a more detailed phenotypic analysis of the same *rlckvi_a2* mutant, focusing on the features that are compromised in the *rpl-4* plants. First, the phyllotactic pattern of siliques was determined by measuring the divergence angles of two successive siliques on the stem (Figure 2). The optimal divergence angle in spiral phyllotaxis is 137.5 degrees [27]. Despite the rarity of individual angles aligning exactly at 137.5 degrees even in wild-type plants, their average tends to approximate this optimal value. In agreement, we found that in wild-type Col-0 plants, the divergence angles showed a rather uniform distribution around the average of 136.98 degrees (Figure 2A). In contrast, the *rpl-4* mutant exhibited a high variation in divergence angles, ranging from 10 to 320 degrees (average 150.98; Figure 2B). The phyllotaxis of the *rlckvi_a2* mutant was disturbed at a lower frequency, resulting in an average degree of 140.73 (Figure 2C). Nevertheless, the frequency of angles falling into the category close to the optimal value (120–149 degrees) decreased from 50% in the wild-type to 35% in the mutant, indicating the likely contribution of the kinase to proper phyllotactic patterning.

**Figure 3:**
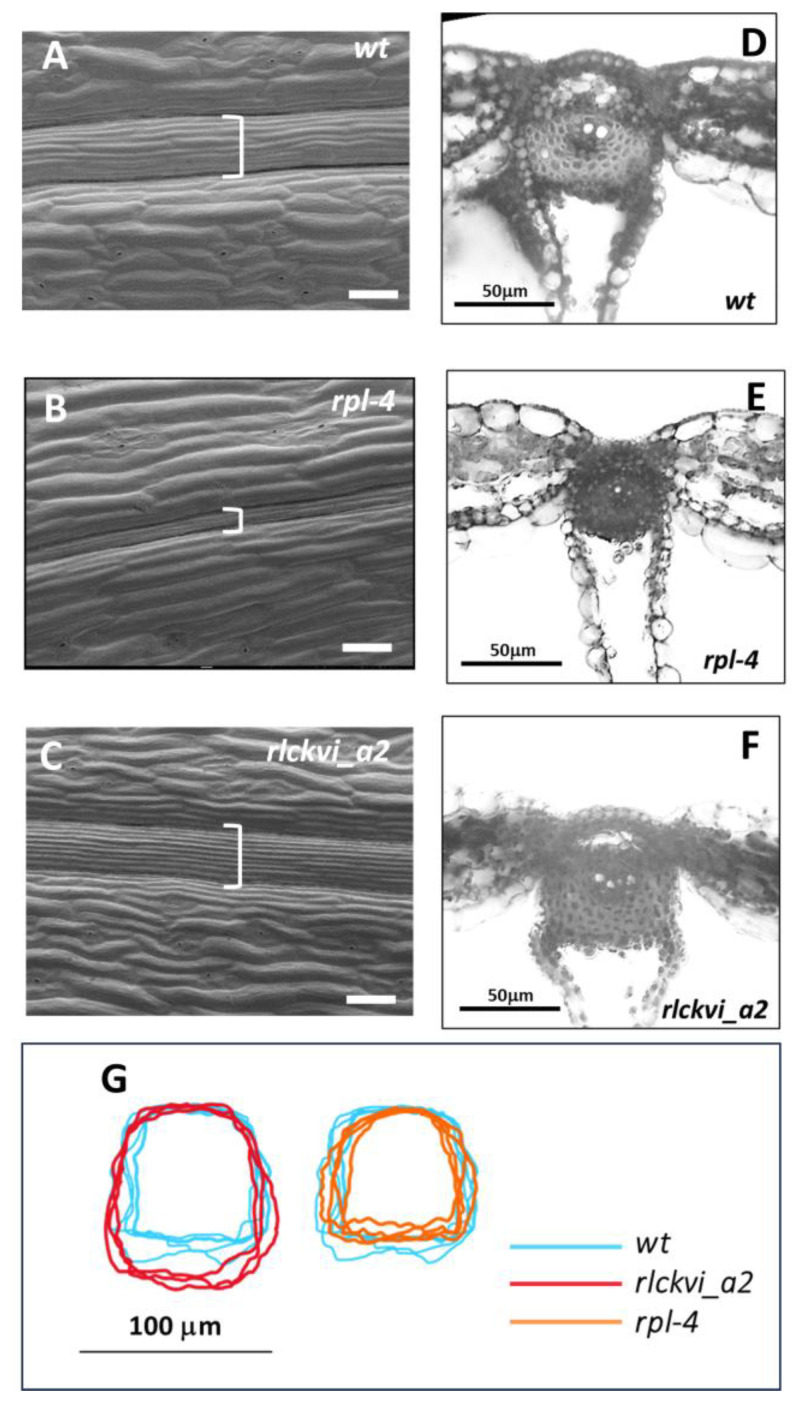
The replum of wild-type (wt), *rpl-4*, and *rlckvi_a2* fruits. Scanning electron microscopy of the fruit surface (**A**–**C**) and cross-sections (**D**–**F**) verified a thinner visible (indicated by ] in (**A**–**C**)) and smaller inner (**D**–**G**) replum for the *rpl-4* mutant compared to the other two lines. Overlaying cross-sections for three replums per line shows that the inner replum of the *rlckvi_a2* mutant is larger than that of the wild-type (**G**), although the visible replum of these two lines is about the same size (**A**,**C**).

As its name indicates, the RPL homeodomain protein is required for replum development (the replum is an abaxial structure of the *Arabidopsis* fruit that remains attached to the plant even after the valves of the mature fruit have fallen) [22]. The identity of the replum is maintained by RPL repressing the valve margin cell fate. Therefore, in *rpl* mutants, the valve margin is extended to the region where the replum would normally develop [22]. In agreement, in mature *rpl-4* fruits, only a narrow cell file could be observed instead of the well-developed replum of the wild-type ones (Figure 3A,B). The mutation in the *RLCKVI_A2* gene did not result in a considerably thinner replum compared to the control (Figure 3C). Microscopical cross-sections of mature fruits verified the compromised development of the smaller replum in the *rpl-4* mutant (Figure 3A–E). In contrast, the inner replum of the *rlckvi_a2* mutant was larger in size compared to the control (Figure 3D,F,G,H). Fluorescent images revealed that the sizes of phloroglucinol-stained cells having lignified walls are larger, but the overall number of these cells is less in the replum of the *rpl-4* and *rlckvi_a2* mutants compared to the wild-type (Figure S2). These lignified cells are present in the separation zone as well as in the inner replum (Figure S2). The cells in the kinase mutant are notably larger in size, thereby leading to the overall enlargement of its inner replum despite a decrease in cell quantity.

**Figure 4:**
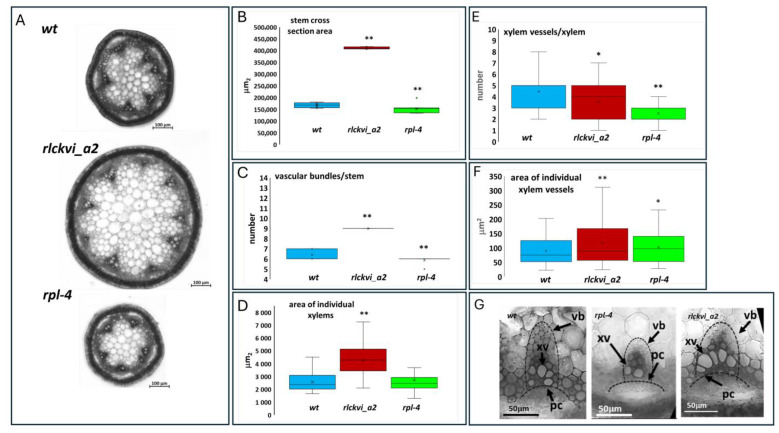
Stem thickness and vascular organization of wild-type (wt), *rlckvi_a2*, and *rpl-4* mutant plants. Cross-sections of the inflorescence stems of the three investigated lines (**A**) were analyzed for stem cross-sectional area (**B**), the number of vascular bundles per stem (**C**), xylem size ((**D**); see dashed lines in (**G**)), the number of xylem vessels per xylem (**E**), and the size of xylem vessels (**F**). Stem parameters were determined for ten plants per line. Xylem parameters were measured for all xylems in four plants per line. The distribution of the measured values is represented as a box plot for each line. Significant differences from the wild-type were determined by Student’s *t*-test (*p* < 0.05 = *; *p* < 0.01 = **). The organization of vascular bundles is exemplified in (**G**). vb—vascular bundle; xv—xylem vessel; pc—arc of procambium.

Investigating the cross-sections of the inflorescence stem verified the roles of both the RPL TF and the RLCKVI_A2 kinase in vascular development and organization. The *rpl-4* mutant has a thinner stem with fewer vascular bundles than the wild-type (Figure 4A,B), in agreement with previous studies [13]. In contrast, the *rlckvi_a2* mutant has a stem cross-sectional area that is more than double that of the wild-type, with approximately 50% more vascular bundles (Figure 4A–C). Nevertheless, the vascular bundles occupy a smaller area of the stem of the *rlckvi_a2* mutant compared to that of the other two lines (Figure S3A), in agreement with the more extended pith of the *rlckvi_a2* stem (Figure S4A).

In the *rpl-4* mutant, the vascular bundles exhibit a flattened arc of procambial cells and a smaller xylem with fewer vessels (Figure 4D–G), as described earlier [13]. In contrast, the line of procambial cells is more dome-shaped, and the vascular bundles have some extra-large xylem vessels in the *rlckvi_a2* mutant compared to the other two lines (Figure 4F,G).

In summary, the *rpl-4* and *rlckvi_a2* mutations both disturb the normal development of the inflorescence stem and the replum. However, the phenotypic changes caused by the two mutations are contrary in many cases (e.g., replum size, stem thickness, number of large xylem vessels).

### 2.3. The Ectopic Expression of the RLCKVI_A2 Gene Interferes with RPL4-Regulated Processes but Cannot Complement the Rpl-4 Mutation

To test the original hypothesis that the lack of *RLCKVI_A2* expression contributes to *rpl-4* phenotypes, we investigated the expression of the 35S promoter-driven *RLCKVI_A2* cDNA in the *rpl-4* mutant as well as in the wild-type and *rlckvi_a2* genetic backgrounds (Figure S4). Plant lines overexpressing the *RLCKVI_A2* cDNA under the control of the 35S promoter were designated as OX (standing for “overexpressor”). The phyllotactic divergence angles were increased by the overexpression of the kinase in the wild-type while hardly affecting those in the *rlckvi_a2* mutant background (Figure 2A,B,D–G). The 35S promoter-driven expression of *RLCKVI_A2* could not complement the *rpl-4* phenotype either (Figure 2C,H,I). A possible reason is that the 35S promoter is unable to provide expression for the *RLCKVI_A2* gene in the same developmental context as its own promoter.

Peaucelle et al. [15] observed the formation of ectopic primordia on *blr6* meristems. We could also observe this defect in a few meristems of the *rpl-4* line. Interestingly, the ectopic expression of the *RLCKVI_A2* gene resulted in the more frequent formation of ectopic organ primordia in the shoot meristem of all investigated genetic backgrounds (37 + −11% in comparison to 10 + −9% in the controls; *n* < 18) (Figure S5). The ectopic activity of the kinase, therefore, might compromise the processes involved in early phyllotactic patterning in the shoot meristem.

**Figure 5:**
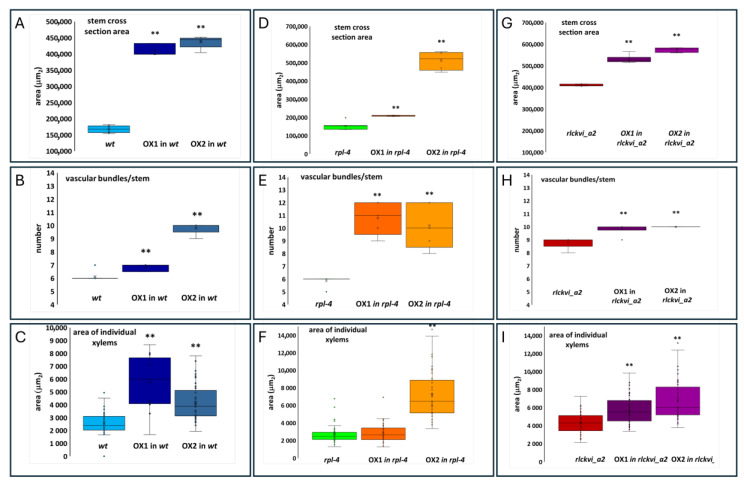
Effect of ectopic RLCKVI_A2 expression on stem thickness and the vasculature. Stem cross-sectional area (**A**,**D**,**G**); the number of vascular bundles per stem (**B**,**E**,**H**); and the average size of xylems (**C**,**F**,**I**) were determined for wild-type (wt; (**A**–**C**)), rpl-4 (**D**–**F**), or rlckvi_a2 (**G**–**I**) plants without and with overexpression (OX1 and OX2) of the *35S:RLCKVI_A2* gene. Ten plants per line were measured. The distribution of the measured values is represented as a box plot for each line. The data of the overexpressor lines (OX1 and OX2) were compared to their respective control using Student’s *t*-test (*p* < 0.01 = **).

**Figure 6:**
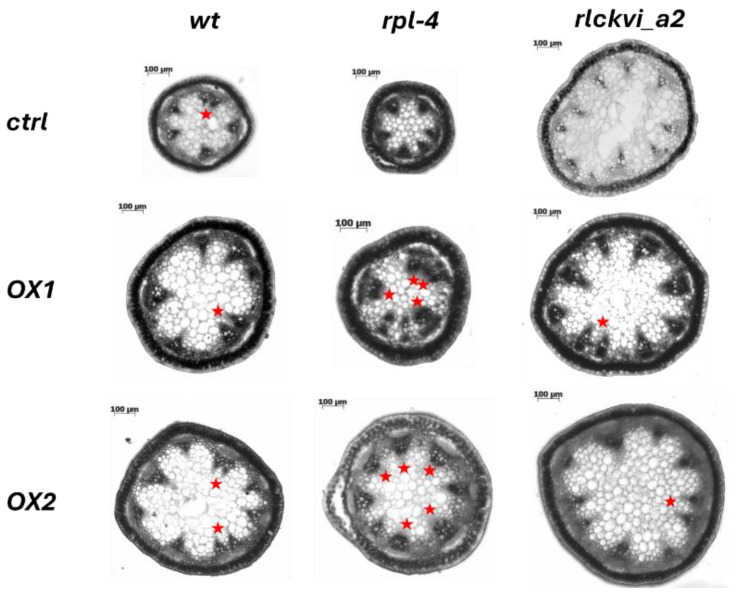
Stem cross-sections of wild-type (wt) and mutant plants (*rpl-4* or *rlckvi_a2*) without and with overexpression (OX1 and OX2) of the *35S:RLCKVI_A2* gene. Red asterisks indicate closely placed/clustered vascular bundles.

The ectopic expression of the *RLCKVI_A2* gene has a profound impact on stem thickness and vascular organization (Figure 5 and Figure 6). The stem cross-sectional area was significantly increased in all three genetic backgrounds (Figure 5A,D,G and Figure 6). Interestingly, the *rlckvi_a2* mutation and the ectopic *RLCKVI_A2* overexpression both increased stem thickness. The effects of the mutation and the ectopic overexpression seem to be independent since they were additive in the *rlckvi_a2* mutant expressing 35S:*RLCKVI_A2* (Figure 5G). This highlights the significance of proper transcriptional control of RLCKVI_A2 expression during stem growth. The ectopic expression of the kinase augmented the number of vascular bundles as well as the size of the xylem in the wild-type such as in the *rlckvi_a2* and *rpl-4* backgrounds (Figure 5B,E,H and Figure 5C,F,I, respectively). It was also observed that the ectopic expression of the kinase resulted in the clustering/fusion of vascular bundles (Figure 6). This could sporadically be observed in the control (non-transgenic) plants as well. In contrast, all the investigated *RLCKVI_A2*-overexpressing plants, regardless of the genetic background, had at least one instance of closely placed or even clustered vascular bundles. The strength of this phenotype seems to depend on stem thickness. A higher number of vascular bundles, caused by the ectopic expression of the kinase, can be placed at more or less regular intervals in the thick stems of *rlckvi_a2* plants. However, this is not possible in thinner wild-type stems, and especially not in the very thin rpl-4 stems. This indicates that the number of vascular bundles and stem thickness are independently regulated by the RLCKVI_A2 kinase.

The ectopic overexpression of *RLCKVI_A2* led to an increase in the number of vessel elements in the xylem of all backgrounds (Figure S6A), although its impact on vessel size remained ambiguous (Figure S6B).

## 3. Discussion

The broad impact of the REPLUMLESS (RPL) TF (also named VAAMANA, PENNYWISE, LARSON, or BELLRINGER) on shoot meristem maintenance, vegetative and reproductive shoot morphogenesis, and organ patterning has been clearly evidenced [11,17,18,23,30,31,32,33,34,35,36,37]. These studies highlighted its complex regulatory interactions with several other TFs. Among others, the direct binding of RPL to the promoter of AGAMOUS, thereby repressing its expression, has been reported [19]. We noticed that the promoter of the *At2G18890 Arabidopsis* gene, which encodes the ROP GTPase-activated receptor-like cytoplasmic kinase, RLCKVI_A2, involved in plant morphogenesis [10], contains nine copies of the same binding elements (Table S1). Since our understanding is limited concerning the downstream cytoplasmic effectors of RPL, we decided to investigate if RPL controls the expression of *RLCKVI_A2*. Introducing the *At2G18890* promoter-controlled GUS marker into the *rpl-4* mutant background revealed that RPL is required for the proper expression of the *RLCKVI_A2* gene that was mainly confined to the shoot apex, vasculature, flower organs, fruit, and seeds (Figure 1). The absence of RPL almost fully abolished the expression of the chimeric construct (Figure 1). Whether this regulation is direct or indirect cannot be known at present. Bencivenga et al. identified potential target genes and binding sites of RPL in *Arabidopsis* inflorescence apices [38]. The *At2G18890* gene was not included in the high confidence list of RPL-regulated genes. Our results indicate under-threshold, tissue-specific, or indirect interactions of RPL and *RLCKVI_A2*.

Nevertheless, we investigated the potential involvement of the RLCKVI_A2 kinase in RPL-controlled processes such as replum development [22], phyllotaxis [15], stem morphogenesis [38], and the differentiation of the vasculature [13]. A detailed analysis of the *rlckvi_a2* T-DNA insertion mutant, previously characterized as a plant line affected in cell/hypocotyl elongation and overall plant growth [10], revealed moderate phenotypic alterations in all of the above processes (Figure 2, Figure 3 and Figure 4). The RLCKVI_A2 kinase belongs to a small protein family with members potentially having overlapping expressions and functions [9] that may explain the relatively weak phenotypes of the mutant. It is of note that although the kinase seems to be involved in similar processes to that of RPL, their mutations sometimes resulted in opposite phenotypes such as in the cross-sectional area of the stem and the replum (Figure 3 and Figure 4). In other cases, they acted in the same direction such as increasing the number of vascular bundles per stem and decreasing the number but increasing the size of lignified cells in the replum or of xylem vessels (Figure 4 and Figure S2). Increasing the number of vascular bundles and indirectly controlling cell wall lignification have been previously described for RPL [18,29].

Although the RLCKVI_A2 kinase is unlikely to be one of the main targets of RPL, we investigated the possibility of *RLCKVI_A2* gene expression using the constitutive 35S promoter to partially complement some phenotypes caused by the *rpl-4* mutation. This assumption was not validated by our observations. Some phenotypes of *rpl-4*, such as phyllotactic aberrations, were not affected (Figure 2), while others, such as the higher number of vascular bundles, were even enhanced (Figure 5E) by ectopic *RLCKVI_A2* expression. Considering that *35S:RLCKVI_A2* expression could not complement even the *rlckvi_a2* mutation but resulted in very similar phenotypes in wild-type, *rpl-4*, or *rlckvi_a2* plants, one can draw the conclusion that the 35S promoter cannot replace the *RLCKVI_A2* promoter. The strong phenotypes caused by ectopic *RLCKVI_A2* expression also highlight the importance of the proper control of *RLCKVI_A2* expression by its own promoter during plant development. The ectopic expression of *RLCKVI_A2* had a profound impact on stem thickness, including the increase in the size of the pith and the number of vascular bundles (Figure 5). Interestingly, *rlckvi_a2* mutation had similar effects on these parameters (Figure 4), and their effects were additive (Figure 5). This suggests that the phosphorylation of some targets by RLCKVI_A2 is a requirement for balanced stem growth and development, but it may have a negative impact outside of the expression domain of the *RLCKVI_A2* gene. There are no known substrates for *Arabidopsis* RLCKVI_A2 at present. However, the mutation of the *RLCK VI_A2* gene has been reported to alter the expression of genes with products that are associated with cell membranes, act at the cell periphery or in the apoplast, and function in cellular transport and/or cell wall reorganization [10]. It is of note that both the RPL and RLCKVI_A2 proteins have been reported to control cell wall organization and cell size, although in an opposite way [10,13,15]. The size of parenchymatic and epidermal cells was observed to be smaller in the case of *RPL* overexpression, while the RLCKVI_A2 kinase was shown to be required for proper cell elongation in the epidermis. Instances where the *rpl-4* and *rlckvi_a2* mutations have opposite phenotypes, such as in stem thickness, may indicate a negative feedback relationship between the transcription factor and the downstream kinase. The RPL transcription factor has been shown to control three-dimensional patterns of cell division in the rib meristem directly repressing organ boundary genes [38]. Even though the *RLCKVI_A2* gene was not identified in a genome-wide screen as a direct target of RPL, it also seems to control stem thickness. It is of note that the *RLCKVI_A2* gene is also expressed in the shoot meristem stem cell niche [39].

## 4. Materials and Methods

### 4.1. Promoter Cloning and Analysis

All plasmids/vectors (except pBSK) were obtained from the *Arabidopsis* Biological Resource Center (http://www.Arabidopsis.org/; accessed on 18 January 2024).

The promoter of the *Arabidopsis* gene *At2G18890* coding for the RLCKVI_A2 kinase was obtained from the F12F24 *Arabidopsis* genomic sequence cloned in the pBleoBAC vector. Based on the sequence information, we identified and isolated the 10,410 bp PstI fragment of the BAC clone, which contained the entire *At2G18890* gene. It was cloned into the pBSK vector (Agilent Technologies Stratagene Products Division, La Jolla, CA, USA) to facilitate further cloning. The HindIII-XbaI fragment carrying 2928 bp upstream and 512 bp downstream of the start codon was ligated to the HindIII-XbaI sites of the binary vector pCXGFP-P ([27]; GeneBank accession FJ905225) from which the BamHI fragment had been previously deleted. Subsequently, the EcoRI-XbaI fragment of the modified pCXGFP-P containing the gene for the GREEN FLUORESCENT PROTEIN was exchanged for the EcoRI-XbaI fragment of the pMDC162 vector [40] encoding the *Esherichia coli* β-glucuronidase (GUS) enzyme. The resulting chimeric gene codes for the first 147 amino acids of the RlCK VI_A2 protein in fusion with the GUS reporter under the control of the *At2G18890* gene promoter. The 147 amino acid-coding sequence was interrupted by the first intron of the *At2G18890* gene. For the map of the final vector molecule, see Figure S1A.

The promoter sequence of the *At2G18890* gene was searched for transcription factor (TF) binding sites at the AtcisDB database (https://agris-knowledgebase.org/AtcisDB; accessed on 18 January 2024).

The cDNA of the *AtRLCKVI_A2* gene was cloned as an EcoRI-XhoI fragment from pET28a:RLCKVI_A2 [4] into the appropriate sites of the pK7F2WG binary Agrobacterium vector in fusion with GFP [41] (for the map, see Figure S1B).

### 4.2. Arabidopsis Transformation

The vector molecules were introduced into *Agrobacterium tumefaciens* (GV3101; pMP90) cells [42] by electroporation [43]. Plant transformation took place in the following way. The 4–6-week-old flowering *Arabidopsis* plants were immersed in an Agrobacterium solution containing 5% sucrose and 0.01% Silwet L77 (Crompton Europe Ltd., Kenneth House, 4 Langley Quay, Slough, Berkshire SL3 6EH, United Kingdom) and then stored covered overnight [44]. This step was repeated after 7–10 days with the new flowers appearing in the meantime to increase the efficiency of the transformation. After that, the plants were allowed to flower, and the mature seeds were collected for the selection of transformants. Selection was performed on ½ AR (half-strength *Arabidopsis* regeneration medium) plates containing 2.2 g/L Murashige Skoog powder (DUCHEFA BIOCHEMIE B.V, Haarlem, The Netherlands), 5 g/L sucrose, and 0.8% agar, supplemented with 20 μg/mL hygromycin for the selection of lines harboring pCXGUS-P, or with 50 μg/mL kanamycin for the selection of pK7F2WG-transformed plants. Plant lines used in further investigations were tested for GFP expression by Western blot analysis (see the next section). 

### 4.3. Western Blot Analysis of GFP:RLCKVI_A2 Protein Abundance

Western blot analysis was carried out according to standard protocols using 1:2000 dilution of anti-GFP rat monoclonal antibody (3H9; ChromoTek GmbH, Planegg/Martinsried, Germany) as the primary and 1:10 000 dilution of horse radish peroxidase-conjugated anti-IgG(H,L) rat antibody (BI2411, Abliance, Compiègne, France) as the secondary antibody. Approximately equal amounts of total protein extracts were loaded on 10% SDS–polyacrylamide gels. After separation, proteins were transferred onto PVDF membranes (Immobilon-P, Merck KGaA, Darmstadt, Germany). The blocking solution was TBS (150 mM NaCl, 50 mM Tris-HCl, pH 7.5) with 0.2% Tween and 5% non-fat milk. Immunoreactive bands were visualized using the Immobilon Western chemiluminescent HRP substrate (WBKLS0500, Merck KGaA) and the iBrightTM CL1500 Imaging System (Thermo Fischer Scientific, Waltham, MA USA). Before performing Western blotting, membranes were stained with a Ponceau S solution (100 mg Ponceau S in 5% acetic acid) as a protein loading control.

### 4.4. GUS Staining

Histochemical analyses were performed with 1-week-old seedlings and 4–8-week-old flowering plants (leaves, flowers, and siliques). GUS activity was detected histochemically by submerging the plant materials in GUS histochemical buffer (100 mM sodium phosphate, 0.5 mM K_3_Fe(CN)_6_, 0.5 mM K_4_Fe(CN)_6_, 10 mM Na_2_-EDTA, 0.1% Triton X-100, and 1 mg/mL 5-bromo-4-chloro-3-indoyl glucuronide (X-gluc)) and incubating them at 37 °C until color developed (12 h ÷ O/N). Samples were cleared with 70% *v*/*v* ethanol and were mounted on microscope slides and observed under a stereomicroscope (Olympus SZX12 Stereozoom Microscope, Evident Corporation, Tokyo, Japan). 

### 4.5. Phyllotactic Pattern Measurements

Plants were grown in greenhouse conditions at 16 h/18 h light/night cycles at 22 °C until siliques were fully developed (developmental stage 8 [28]). Stems were then cut right above rosette leaves; photos of all stems were taken, and divergence angles were measured between the insertion points of two successive siliques according to Peaucelle A et al. [15]. The top 5 cm of the stem was not assessed, as elongation was incomplete. The phyllotactic orientation was set to the direction giving the smallest average divergence angle. For graphical representation, divergence angles between two successive siliques on the stem were allocated into twelve 30° classes, and the percentage of total measurements (n) falling into each class was visualized.

### 4.6. Scanning Electron Microscopy (SEM)

For SEM measurements, shoot apical meristems were collected when plants reached the bolting phase with 1–2 cm long stems, while replums were collected when stems were fully developed. Plant samples were vacuum-infiltrated and then fixed in a 2.5% glutaraldehyde-containing phosphate buffer (pH 7.4) for 3 h or overnight, dehydrated in aqueous solutions of increasing ethanol concentrations, critical-point-dried, covered with 15 nm gold using Quorum Q150T ES sputter (Quorum Technologies, Laughton, East Sussex, UK), and observed in a JEOL JSM-7100F/LV scanning electron microscope (JEOL Europe BV, Nieuw-Vennep, The Netherlands) using 3–5 kV accelerating voltage.

### 4.7. Preparation and Microscopy of Cross-Sections of Arabidopsis Siliques and Inflorescence Stems

Small (generally 0.5 cm long) pieces of samples derived from *Arabidopsis* siliques, or the axes of the inflorescences were fixed in 4% (*w*/*v*) paraformaldehyde according to the protocol of [45]. After fixation, silique or inflorescence stem samples were washed in distilled water and embedded in 5% agarose based on [46]. Then, 100 μm thick cross-sections were prepared using a vibratome (Microm HM 650 V, Thermo Fischer Scientific). The sections were placed on a microscopic slide with a drop of water and were stained with toluidine blue [TB; 0.02% (*w*/*v*)] to stain the cell walls [47]. To visualize lignified cells in the tissue samples, sections were also incubated in phloroglucinol–HCl (Wiesner’s reagent) [48]. The sections were observed with a light microscope and an inverted fluorescent microscope (Zeiss Axiovert 200 M, Carl Zeiss, Oberkochen, Germany) equipped with a digital camera (AxiocamHR, HQ CCD, Carl Zeiss), applying filter set 9 (exc.: 450–490 nm, em.:515–∞ nm) and filter set 49 (exc.: 365 nm, em.: 445/50 nm).

### 4.8. Image Processing, Statistics, and Reproducibility

Scanning electron microscopic images as well as fluorescent and light microscopic images were analyzed with the aid of ImageJ software (version 1.54h) [49]. Statistical analyses were carried out using the Excell software v. 2021 (Microsoft Corporation, Redmond, WA, USA), and treated statistically by Student’s *t*-test pairwise comparing the treated and control samples. Statistical significance was deemed significant at a minimum threshold of *p* < 0.05. Non-quantitative experiments were performed at least three times with independent biological samples and were included if produced similar results.

## 5. Conclusions

The RPL TF is required for the tissue-specific expression of the ROP GTPase-activated RLCKVI_A2 kinase gene. Although previous studies could not identify RLCKVI_A2 as a direct target of RPL, the kinase and the TF seem to influence similar processes, including stem growth, phyllotaxis, and replum size. Our results highlight that the expression of the kinase in cells/tissues where it is not normally expressed alters shoot/fruit morphogenesis and vascular differentiation. This highlights the importance of proper developmental control of its promoter, for which the RPL TF might be partly responsible.

## Data Availability

The original contributions presented in the study are included in the article/Supplementary Materials, further inquiries can be directed to the corresponding author.

## Supplementary Materials

The following supporting information can be downloaded at: https://www.mdpi.com/article/10.3390/ijms25148001/s1.
