# Supplementary material for: The REPLUMLESS Transcription Factor Controls the Expression of the RECEPTOR-LIKE CYTOPLASMIC KINASE VI_A2 Gene Involved in Shoot and Fruit Patterning of Arabidopsis thaliana
Source: Int J Mol Sci. 2024 Jul 22;25(14):8001. doi: 10.3390/ijms25148001 (PMC11277442; doi:10.3390/ijms25148001)
Supplement: Supplementary file 1 [file ijms-25-08001-s001.zip › ijms-3115640-supplementary.pdf]

Supplementary Materials

Figure S1:

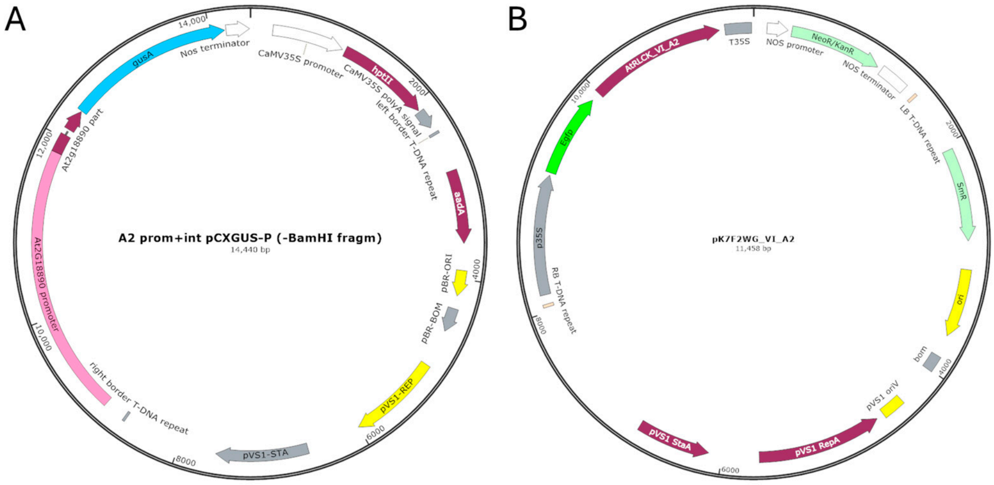

FigS. 1. Vector maps. A) Vector to analyse the expression of the At2G18890 promoter-driven -GLUCURONIDASE (GUS) reporter gene in transgenic Arabidopsis plants. B) Vector to overexpress the RLCKVI_A2 kinase fused to an N-terminal GFP-tag under the control of the constitutive 35S promoter.

Figure S2:

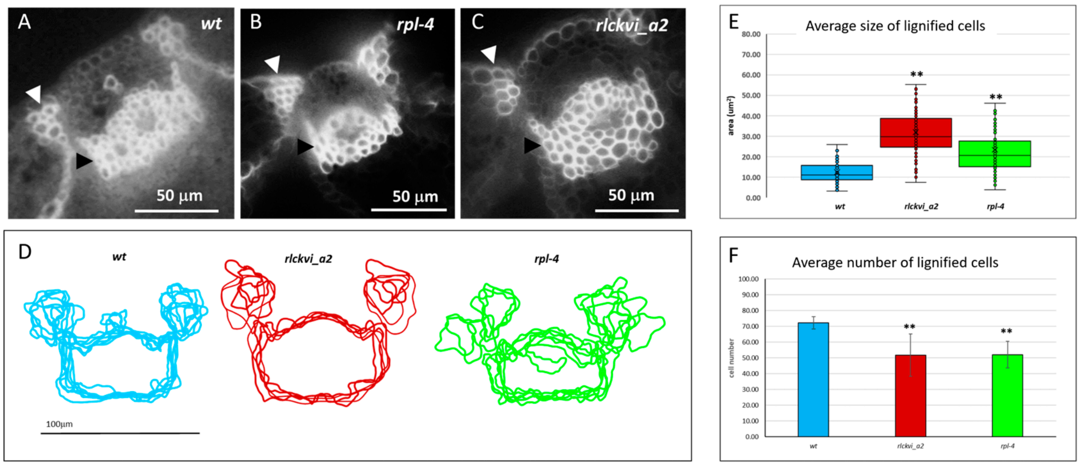

FigS. 2. Lignified cells in the replum of the wild type (wt), rlckvi_a2 and rpl-4 mutants. Lignified cell walls were visualized by fluorescence microscopy after phloroglucinol-staining (A-C). The borders of the areas occupied by lignified cells in five replums of each of the three lines are also shown (D). Size (E) and number per replum (F) were measured for ten replums of each line. The distribution of the measured values is represented as a box plot for each line. The data of the mutants were compared to those of the wild type using Student's t-test ($p < 0.05$ = *; $p < 0.01$ =**).

Figure S3:

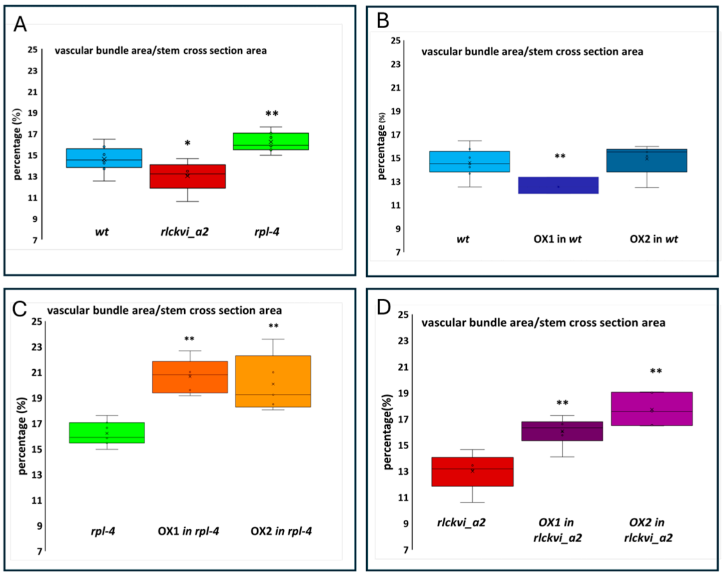

FigS. 3. The ratio of the stem cross-sectional area and the area occupied by vascular bundles within it. Comparison of the wild-type with rlckvi_a2 and rpl-4 mutants (A), and the same three lines expressing or not expressing the 35S promoter-driven RLCKVI_A2 gene (B-D). Ten plants were investigated per line. The distribution of the measured values is represented as a box plot for each line. Significant differences from the wild-type and/or control lines were determined by Student's t-test ($p < 0.05$ = *; $p < 0.01$ = **).

Figure S4:

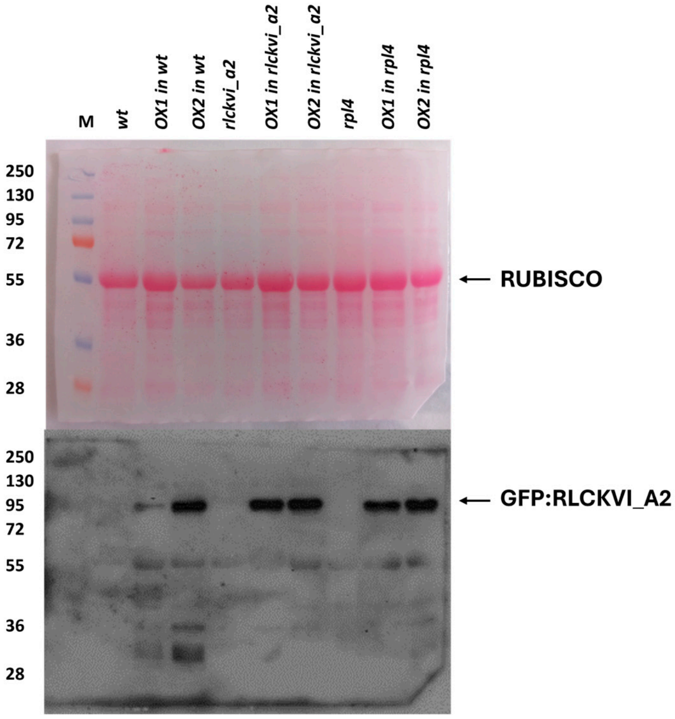

FigS. 4. Expression of the 35S promoter-controlled RLCKVI_A2 gene in the investigated wild-type(wt), *rlckvi_a2* and *rpl-4* mutant transgenic plant lines. Non-transgenic plants were used as controls, along with two independent transformants overexpressing the kinase gene (OX1 and OX2), for each of the three lines. The upper image shows the Ponceau S-stained membrane, highlighting the 56 kDa RUBISCO subunit as a protein loading control. The image below shows the result of the Western analysis highlighting the 83 kDa GFP_RLCKVI_A2 protein detected by an anti-GFP antibody. M = molecular mass standard

Figure S5:

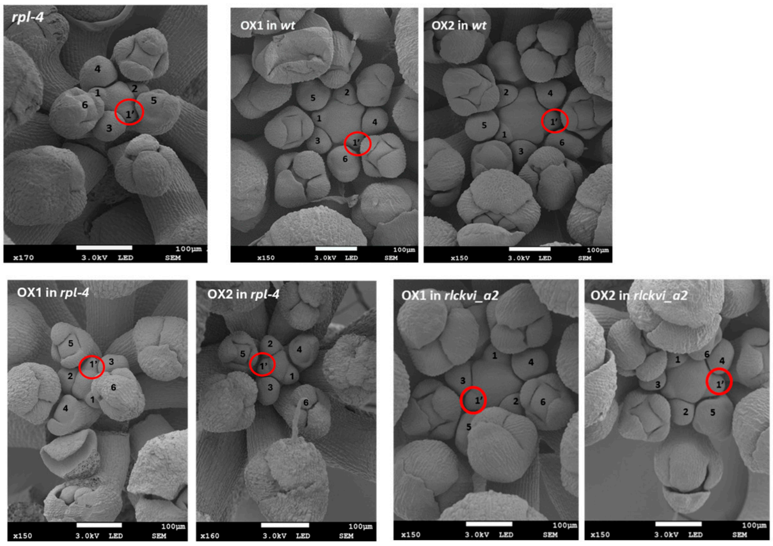

FigS. 5. Examples of ectopic leaf primordium formation in the various investigated genetic backgrounds. Leaf primordia are numbered based on their order of appearance, where 1 is the youngest primordium. 1' labels the ectopic primordia, which are also encircled in red. wt – wild type; OX1 and OX2 stands for two independent lines overexpressing the RLCKVI_A2 kinase.

Figure S6:

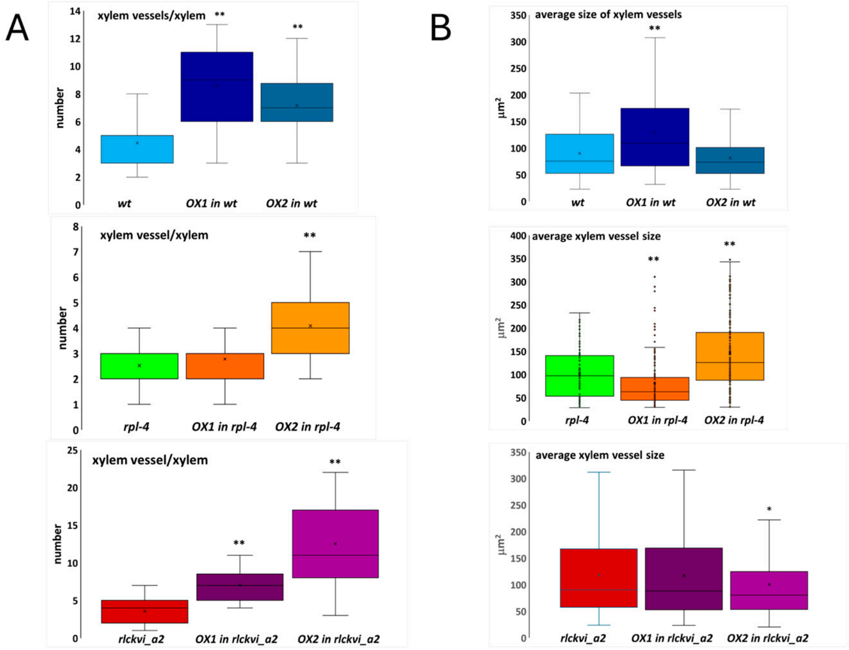

FigS. 6. The effect of ectopic RLCKVI_A2 expression on xylem vessel number (A) and size (B). These paremeters have been determined for wild-type (wt), rpl-4, or rlckvi_a2 plants without and with overexpression (OX1 and OX2) of the 35S:RLCKVI_A2 gene, as indicated. All xylems were investigated for four plants per line. The distribution of the measured values is represented as a box plot for each line. The data of the overexpressor lines (OX1 and OX2) were compared to their respective control using Student's t-test ($p < 0.05$ = *; $p < 0.01$ =**).
